# Supplementary figures and images for: BOXR1030, an anti-GPC3 CAR with exogenous GOT2 expression, shows enhanced T cell metabolism and improved anti-cell line derived tumor xenograft activity
Source: PLoS One. 2022 May 4;17(5):e0266980. doi: 10.1371/journal.pone.0266980 (PMC9067639; doi:10.1371/journal.pone.0266980)

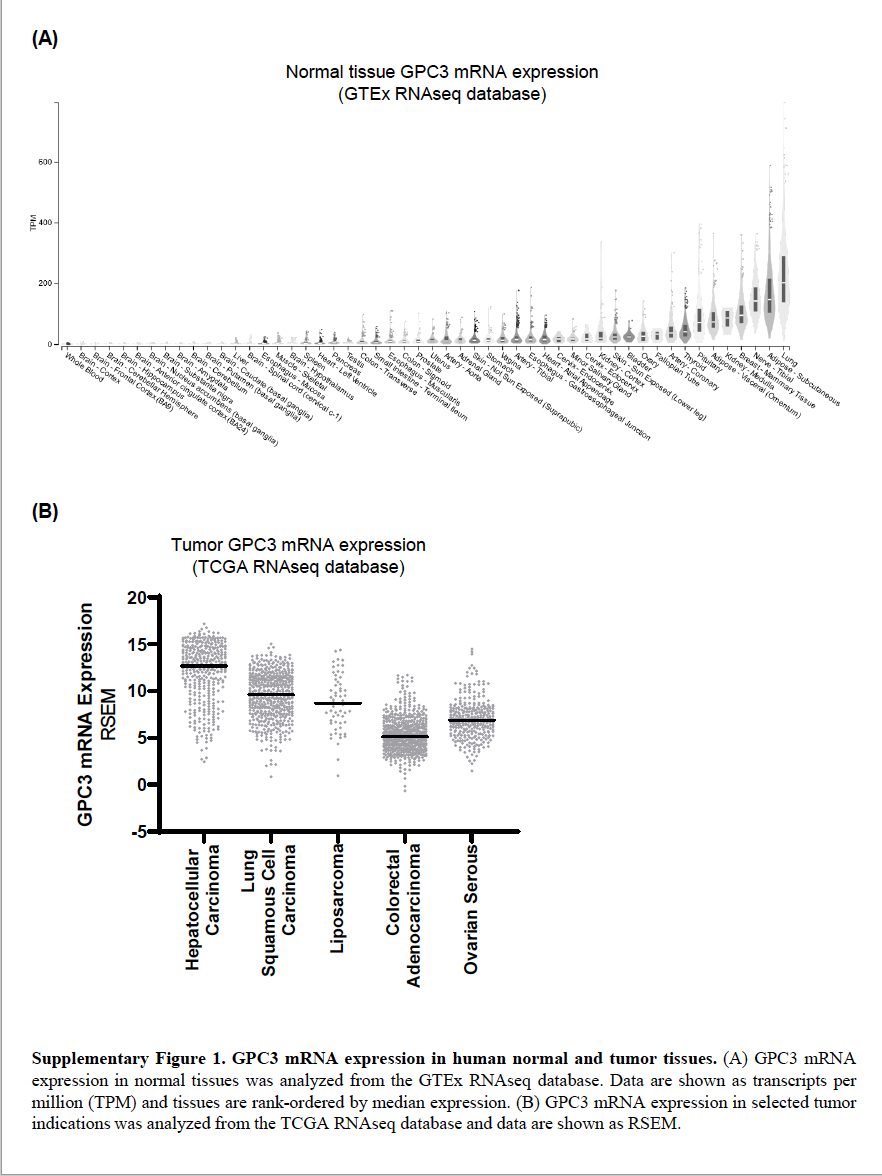

Supplement: S1 Fig — (A) GPC3 mRNA expression in normal tissues was analyzed from the GTEx RNAseq database. Data are shown as transcripts per million (TPM) and tissues are rank-ordered by median expression. (B)GPC3 mRNA expression in selected tumor indications was analyzed from the TCGA RNAseq database and data are shown as RSEM. (JPG) [file pone.0266980.s001.jpg]

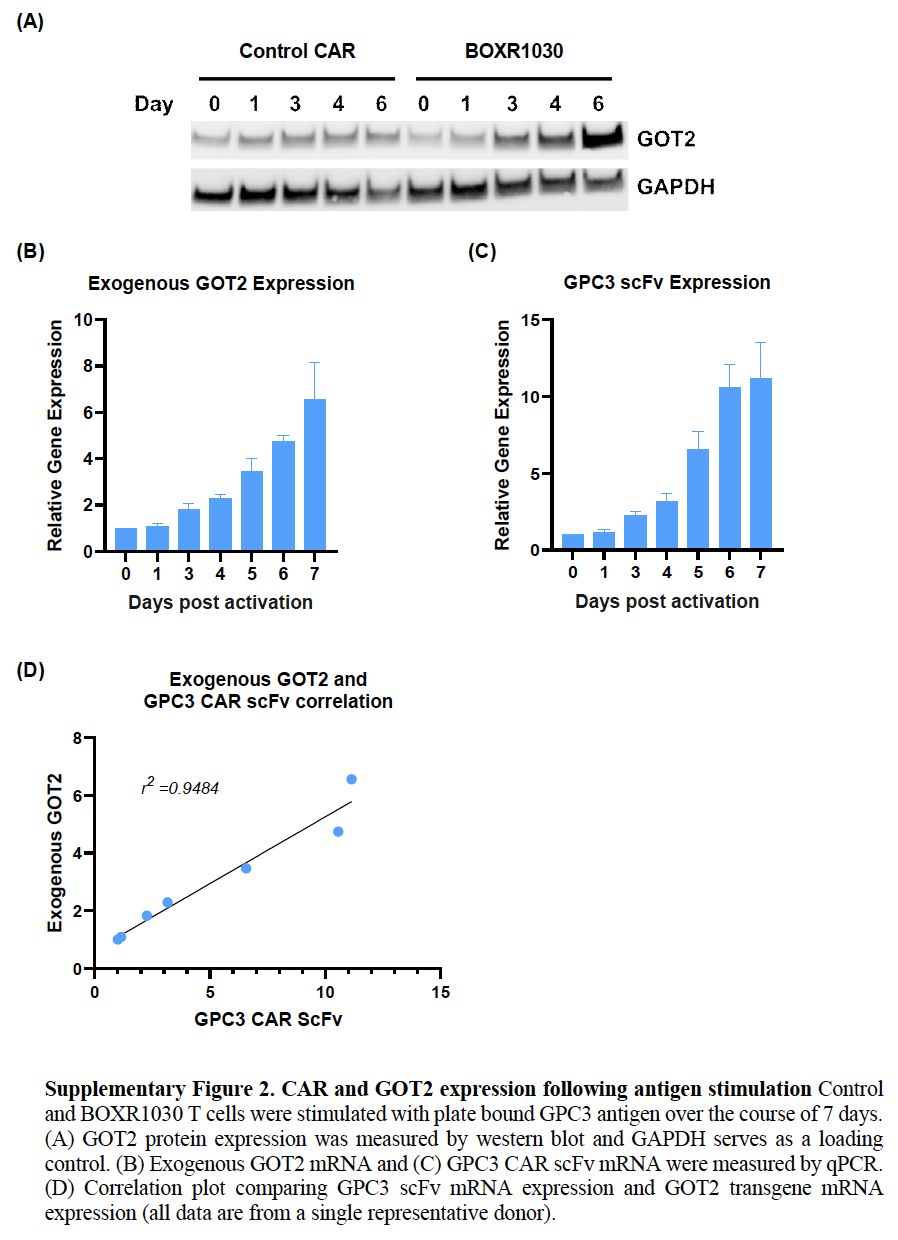

Supplement: S2 Fig — Control and BOXR1030 T cells were stimulated with plate bound GPC3 antigen over the course of 7 days. (A) GOT2 protein expression was measured by western blot and GAPDH serves as a loading control. (B) Exogenous GOT2 mRNA and (C) GPC3 CAR scFv mRNA were measured by qPCR. (D) Correlation plot comparing GPC3 scFv mRNA expression and GOT2 transgene mRNA expression (all data are from a single representative donor). (JPG) [file pone.0266980.s002.jpg]

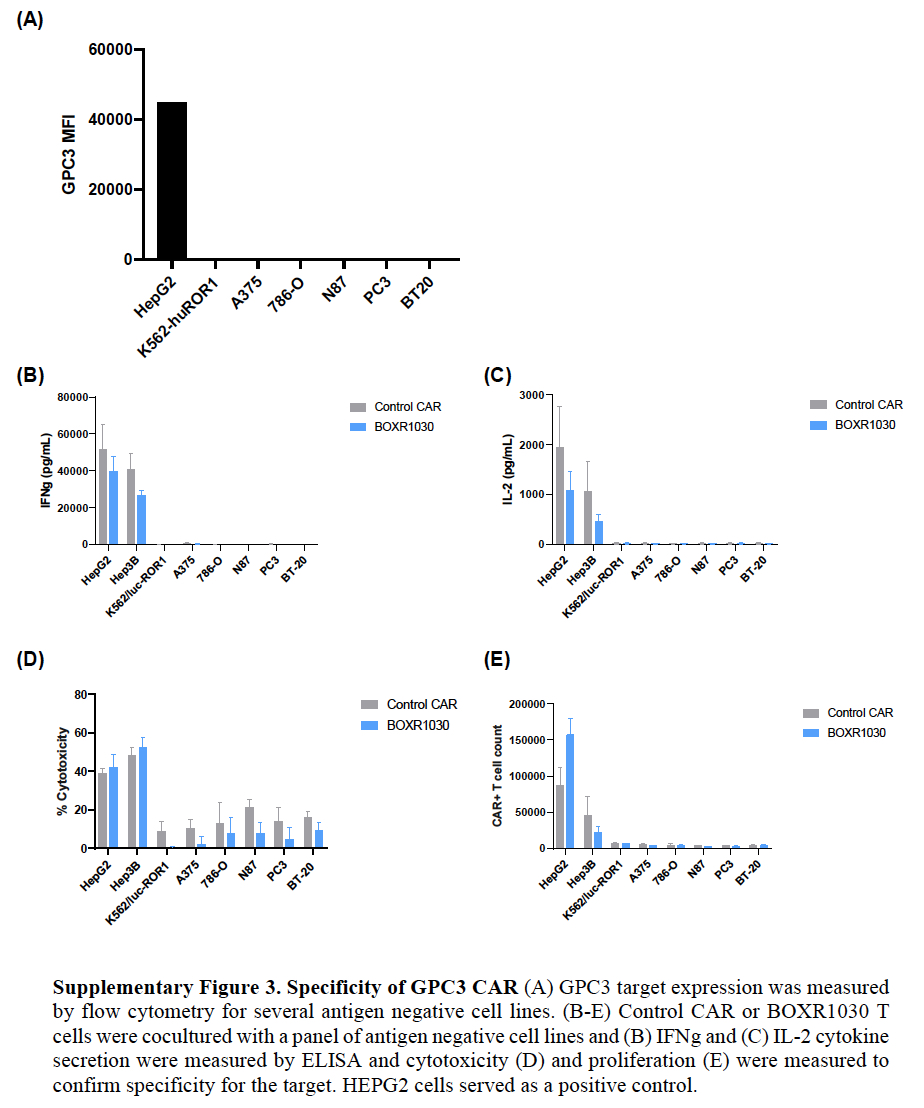

Supplement: S3 Fig — (A) GPC3 target expression was measured by flow cytometry for several antigen negative cell lines. (B-E) Control CAR or BOXR1030 T cells were cocultured with a panel of antigen negative cell lines and (B) IFNg and (C) IL-2 cytokine secretion were measured by ELISA and cytotoxicity (D) and proliferation (E) were measured to confirm specificity for the target. HEPG2 cells served as a positive control. (JPG) [file pone.0266980.s003.jpg]

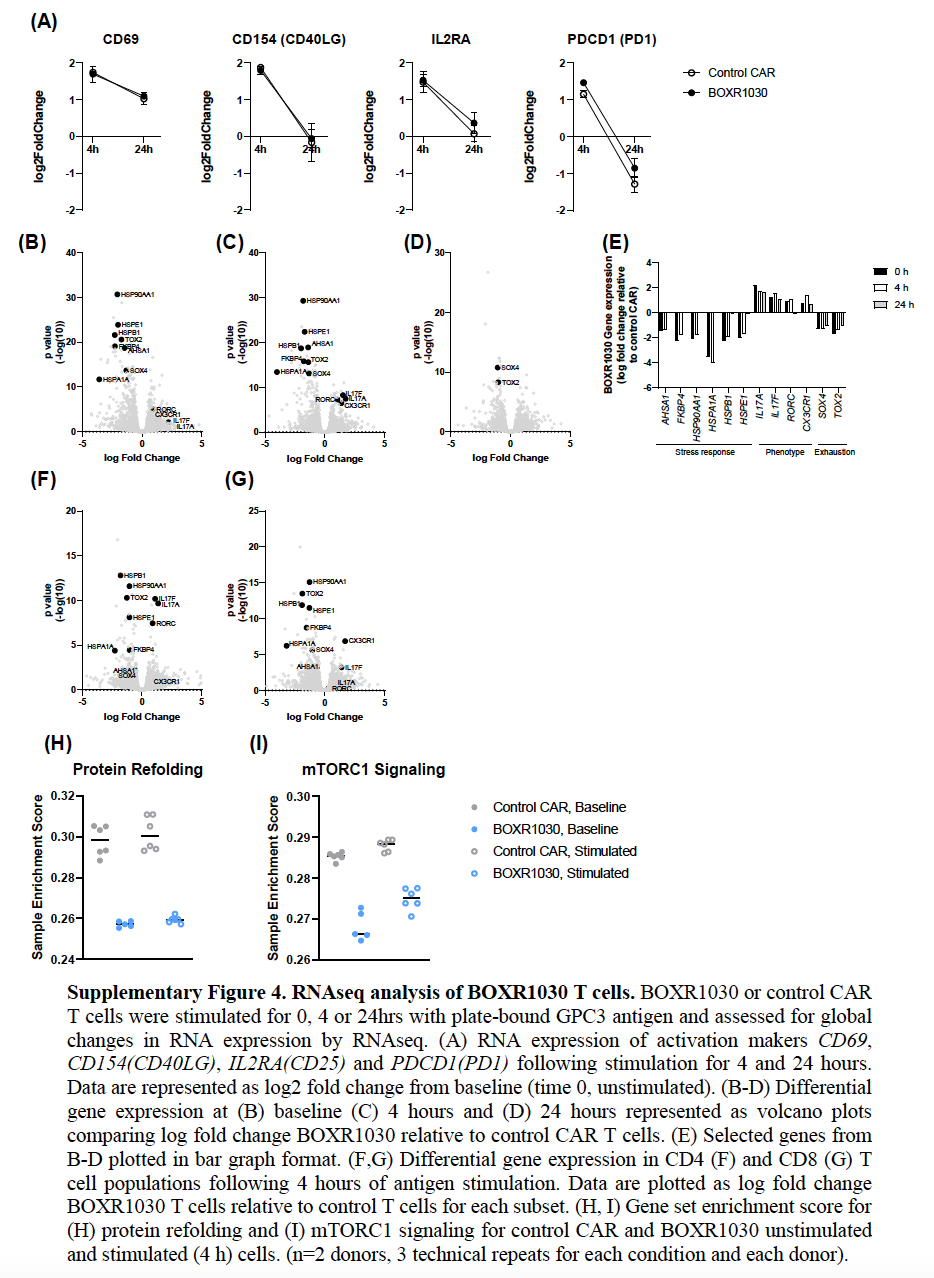

Supplement: S4 Fig — BOXR1030 or control CAR T cells were stimulated for 0, 4 or 24hrs with plate-bound GPC3 antigen and assessed for global changes in RNA expression by RNAseq. (A) RNA expression of activation makers CD69, CD154(CD40LG), IL2RA(CD25), and PDCD1(PD1) following stimulation for 4 and 24 hours. Data are represented as log2 fold change from baseline (time 0, unstimulated). (B-D) Differential gene expression at (B) baseline (C) 4 hours and (D) 24 hours represented as volcano plots comparing log fold change BOXR1030 relative to control CAR T cells. (E) Selected genes from B-D plotted in bar graph format. (F,G) Differential gene expression in CD4 (F) and CD8 (G) T cell populations following 4 hours of antigen stimulation. Data are plotted as log fold change BOXR1030 T cells relative to control T cells for each subset. (H, I) Gene set enrichment score for (H) protein refolding and (I) mTORC1 signaling for control CAR and BOXR1030 unstimulated and stimulated (4 h) cells. (n = 2 donors, 3 technical repeats for each condition and each donor). (JPG) [file pone.0266980.s004.jpg]
